# Congenital thrombocytopenia associated with *GNE* mutations in twin sisters: a case report and literature review

**DOI:** 10.1186/s12881-020-01163-2

**Published:** 2020-11-16

**Authors:** Xin Li, Ying Li, Min Lei, Jing Tian, Zuocheng Yang, Shoujin Kuang, Yanjuan Tan, Tao Bo

**Affiliations:** 1 Department of Pediatrics, The Third Xiangya Hospital, Central South University, Tongzipo Road NO.138, Changsha, 410013 China; 2 Chengdu Women’s and Children’s Central Hospital, School of Medicine, University of Electronic Science and Technology of China, Chengdu, 611731 Sichuan China

**Keywords:** Congenital thrombocytopenia, UDP-N-acetyl-glucosamine 2-epimerase/N-acetylmannosamine kinase (*GNE*), Sialic acid, Neonates, Twins

## Abstract

**Background:**

Neonatal thrombocytopenia is common in preterm and term neonates admitted to neonatal intensive care units. The etiology behind neonatal thrombocytopenia is complex. Inherited thrombocytopenia is rare and usually results from genetic mutations.

**Case presentation:**

Here we report a case of twins with severe inherited thrombocytopenia presented in the neonatal period who were shown to be compound heterozygotes for 2 UDP-N-acetylglucosamine 2-epimerase (*GNE*) gene mutations, c.1351C > T and c.1330G > T, of which c.1330G > T is a novel mutation.

**Conclusion:**

These two *GNE* mutations may help in the diagnosis and management of thrombocytopenia diagnosed in neonates.

## Background

Thrombocytopenia is defined as a platelet count < 150 × 10^9^/L and is the most frequent hematological disorder observed in neonates admitted to the neonatal intensive care unit (NICU). This disorder has an incidence between 18 and 35% [1] It is known that reduced production of platelets induces thrombocytopenia, whose heritable forms are frequently caused by genes that regulate megakaryocytic differentiation and/or platelet production [2] With the development of diagnostic methods, neonatal thrombocytopenia, especially the congenital type, was linked to genetic mutations.

UDP-N-acetyl-glucosamine 2-epimerase/N-acetylmannosamine kinase (*GNE*), a key enzyme of sialic acid biosynthesis, initiates and regulates the biosynthesis of N-acetylneuraminic acid [3] Mutations in *GNE* have been identified in both hereditary inclusion body myopathy and Nonaka myopathy, which is also termed as distal myopathy with rimmed vacuoles (DMRV; MIM#605820) [2, 4] In recent years, the relationship between *GNE* mutations and congenital thrombocytopenia has been reported [3, 5–8] Here, we report a case of twin sisters who were shown to have novel combined heterozygous *GNE* mutations and diagnosed with refractory thrombocytopenia.

## Case report

**Fig. 1:**
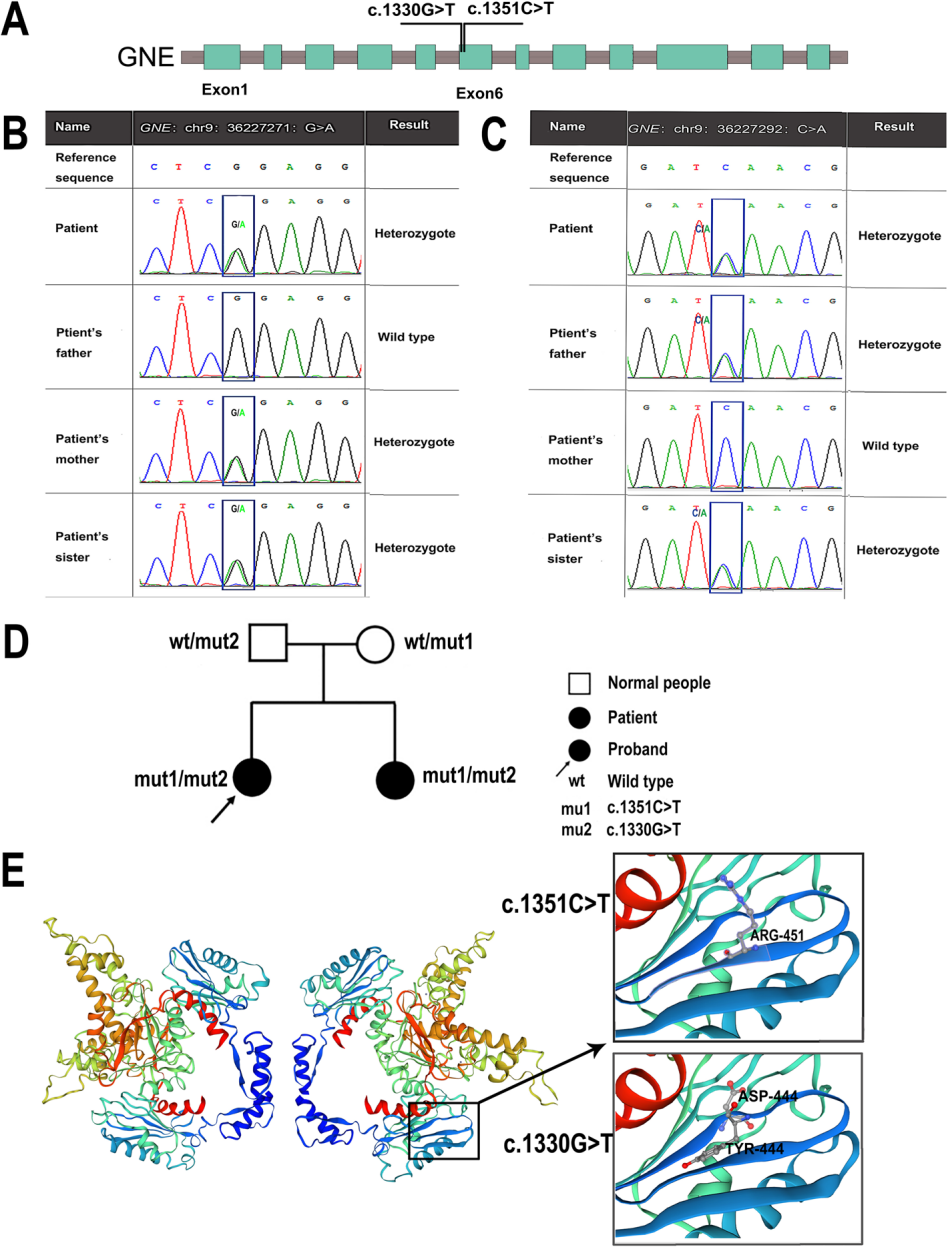
Sequencing and a genogram of the Trio. **a**:Schematic diagram of GNE exons. Mutation positions were labeled with black lines. **b**: The c.1351C > T mutation. This mutation is a non-sense mutation, where arginine (Arg) mutates to a termination codon, which is inherited from the mother. **c**: The c.1330G > T mutation. This mutation is inherited from the father and is a missense mutation, resulting when aspartic acid (Asp) mutates to tyrosine (Tyr). **d**: Parents are carriers of each mutation and show no phenotypes. The twins simultaneously inherited two mutations from their parents and presented with refractory thrombocytopenia several days after birth. **e**: 3D homology model changes induced by the c. 1330G > T and c.1351C > T mutations

A 22-day-old female newborn exhibited a platelet count of 1 × 10^9^/L and showed detectable traces of blood in her stools. At the same time, her twin sister also had a platelet count of 6 × 10^9^/L. The twins were born 37 3/7 weeks of gestational through cesarean section to a mother who denies unfavorable obstetric or medical histories. Moreover, there was no history of thrombocytopenia in the family. When the newborns were admitted to the NICU, vital signs and physical examinations appeared normal. Blood laboratory studies showed severe thrombocytopenia and mild anemia in the twins. The size of their peripheral blood platelets was shown to range from normal to large and their mean platelet volume was 10.6–13.2 fl (reference range: 6.5-12 fl). Routine urine, stool, liver function and renal function tests all appeared as normal. TORCH- and platelet-associated antibodies were negative. Megakaryocytes in the bone marrow showed normal morphology, but their maturation was hindered. Whole exon sequencing (WES) analysis revealed novel compound heterozygous mutations in exon 6 of the *GNE* gene (Fig. 1a), including c.1351C > T, (p. Arg451*) and c.1330G > T, (p. Asp444Tyr) (Fig. 1b, c). No other gene mutations related to thrombocytopenia were identified. A genogram directly confirmed that the twins were compound heterozygotes for these mutations (Fig. 1d). In the Genome Aggregation Database (gnomAD), the occurrence frequency of the two mutations was found to be 4/251452 (c.1351C > T) and 1/251452 (c.1330G > T). The c. 1330G > T was found to be a novel mutation. Despite being reported in HGMD as described by Tomimitsu [9] et al and Mori-Yoshimura [10] et al, c.1351C > T mutations associated with thrombocytopenia are first reported here. The p. Arg451* based reference sequence NM_005476.6 is a non-sense mutation. Furthermore, mutation pathogenicity prediction software, including Sorting Intolerant From Tolerant (SIFT), Polyphen2, LRT, MutationTaster, PredictSNP (https://loschmidt.chemi.muni.cz/predictsnp/) and FATHMM, predicted that the c.1330G > T mutation was harmful and destabilizing. Homology modeling was performed for the wild-type or mutant proteins using the SWISS-MODEL server (https://swissmodel.expasy.org/). As shown in Fig. 1e, c.1351C > T results in the deletion of 8 amino acids starting at position 451, whereas c.1330G > T results in an Asp to Tyr mutation at position 444. Therefore, the twins were diagnosed with congenital thrombocytopenia associated with *GNE* mutations.

The twins received platelet and intravenous immunoglobulin (IVIG) transfusions soon after admission to the NICU given their severe thrombocytopenia, but their baseline platelet counts remained as less than 10 × 10^9^/L. After a 3-month follow-up, the twins did not show bleeding or signs of muscle wasting.

## Discussion and conclusion

The human *GNE* gene (NM_001128227.2), localized on chromosome 9p13.3, consists of 14 exons and encodes 724 amino acids [3, 5, 6, 11] The protein encoded by this gene is a bifunctional enzyme that initiates and regulates the biosynthesis of N-acetylneuraminic acid (NeuAc), which is a precursor of sialic acid [12]^.^

Mutations in the *GNE* gene decreases its enzymatic activity and affects the two initial steps in sialic acid biosynthesis. As a result, sialic acid production decreases and consequently, sialylation, or the incorporation of sialic acid to glycoproteins and glycolipids, also decreases [13] Hyposialylation was previously identified in mouse models of *GNE* myopathy [14] More than 10 *GNE* gene mutations have been reported in human DMRV. However, the exact mechanisms behind *GNE* defects leading to DMRV are still not fully understood.

As a major determinant of cell surface sialylation in human hematopoietic cell lines and a critical regulator of cell surface adhesion molecules [15], *GNE* is also expressed in bone marrow. Sialic acid is known to play significant roles in platelet functions and is located in the platelet membrane, forming during glycosylation. Several groups have shown that platelets deficient in sialylation are removed from circulation, resulting in shortened circulation lifetimes and thrombocytopenia [16]

In 2014, two families diagnosed with DMRV associated with thrombocytopenia were reported. Izumi et al [2] reported that two adult siblings diagnosed with DMRV and thrombocytopenia harbored two compound heterozygous *GNE* mutations, p.V603L and p.G739S. Zhen et al [17] reported that two adult siblings in a DMRV family exhibited thrombocytopenia and showed compound heterozygous *GNE* mutations including a missense mutation c.649 T > C, (p.Y217H) and a frameshift mutation c.1543–1544 del GA, (p.D515Qfs*2). These patients showed mild-to-moderate thrombocytopenia and no obvious bleeding.

In 2018, it was reported that *GNE* mutations cause thrombocytopenia without associated myopathy. Futterer et al [8] reported that two cousins in a consanguineous family manifested moderate to severe bleeding associated with severe thrombocytopenia and required regular platelet transfusions. In addition, homozygous mutations c. 1246 G > A, (p. G416r) in the *GNE* gene were detected. Revel-Vilk et al [18] summarized nine patients from three families all exhibiting thrombocytopenia and mild to moderate hemorrhagic tendencies. All of these individuals contained compound homozygous or heterozygous mutations in *GNE*, of which eight of the nine patients in showed no evidence of myopathy and one patient with neuromuscular symptoms showed a muscle biopsy inconsistent with *GNE* myopathy.

In the case of the twins described here, refractory severe thrombocytopenia was identified in the neonatal period and two novel compound heterozygous *GNE* mutations, p.Arg451* and p.Asp444Tyr, were detected using whole-exome sequencing. Compared with other reports, these twins are some of the youngest reported and their baseline platelet counts were of the lowest values when they were diagnosed. Consistent with other reports [17, 19, 20], mutations in *GNE* associated with thrombocytopenia were within the N-acetylmannosamine (ManNAc) kinase domains of the *GNE* protein. Thus, this domain may be an interesting site for further investigation related to congenital thrombocytopenia associated with *GNE* mutations.

In summary, in this report, we describe twins with two novel compound heterozygous *GNE* mutations, including p.Arg451* and p.Asp444Tyr, who manifested only severe thrombocytopenia without myopathy. Yet the role of *GNE* protein in the synthesis and functions of platelet requires further investigation.

## Data Availability

The datasets analysed during the current study are available in the 360 medical repository(https://yunpan.360.cn/surl_yPErJZuASnH). NM_005476.6(genebank https://www.ncbi.nlm.nih.gov/nuccore/NM_005476.6). NM_001128227.2(genebank https://www.ncbi.nlm.nih.gov/nuccore/NM_001128227.2).

## Abbreviations

*GNE*: UDP-N-acetyl-glucosamine 2-epimerase/N-acetylmannosamine kinase
NeuAc: N-acetylneuraminic acid
ExAC: Exome Aggregation Consortium
gnomAD: Genome Aggregation Database
SIFT: Sorting Intolerant From Tolerant
